# Major role for mRNA stability in shaping the kinetics of gene induction

**DOI:** 10.1186/1471-2164-11-259

**Published:** 2010-04-21

**Authors:** Ran Elkon, Eitan Zlotorynski, Karen I Zeller, Reuven Agami

**Affiliations:** 1 Division of Gene Regulation, The Netherlands Cancer Institute. Plesmanlaan 121, 1066 CX Amsterdam; The Netherlands; 2 Division of Hematology, Department of Medicine, Johns Hopkins School of Medicine, Baltimore, MD, USA; 3 Center for Biomedical Genetics, The Netherlands

## Abstract

**Background:**

mRNA levels in cells are determined by the relative rates of RNA production and degradation. Yet, to date, most analyses of gene expression profiles were focused on mechanisms which regulate transcription, while the role of mRNA stability in modulating transcriptional networks was to a large extent overlooked. In particular, kinetic waves in transcriptional responses are usually interpreted as resulting from sequential activation of transcription factors.

**Results:**

In this study, we examined on a global scale the role of mRNA stability in shaping the kinetics of gene response. Analyzing numerous expression datasets we revealed a striking global anti-correlation between rapidity of induction and mRNA stability, fitting the prediction of a kinetic mathematical model. In contrast, the relationship between kinetics and stability was less significant when gene suppression was analyzed. Frequently, mRNAs that are stable under standard conditions were very rapidly down-regulated following stimulation. Such effect cannot be explained even by a complete shut-off of transcription, and therefore indicates intense modulation of RNA stability.

**Conclusion:**

Taken together, our results demonstrate the key role of mRNA stability in determining induction kinetics in mammalian transcriptional networks.

## Background

mRNA levels in cells are determined by the relative rates of RNA production and degradation. Transcript levels at steady state therefore reflect equilibrium of RNA synthesis and decay. Gene expression microarrays, and more recently RNA deep-sequencing, are valuable means for genome-wide profiling of the cellular transcriptome and its modulation during normal development and in response to external perturbations. Standard microarray analyses on total cellular RNA provide a measure of mRNA abundance but cannot discriminate whether changes are due to alterations in RNA transcription or decay. New techniques have been developed to allow the measurement of actual rates of transcript production. When these are carried out in parallel to recording overall RNA abundance, they can reveal the relative contribution of alterations in gene transcription and mRNA stability to the observed net change in RNA abundance [1,2].

Despite the above, to date, the contribution of RNA degradation to global changes in cellular transcriptome was largely overlooked. This was mostly due to the fact that most microarray studies, explicitly or implicitly, ascribed alterations in RNA levels to correlated changes in gene transcription. However, several pioneering studies have shed light on the critical role that modulation of mRNA stability plays in the regulation of cellular transcriptome in response to various stresses [3-7].

**Figure 1:**
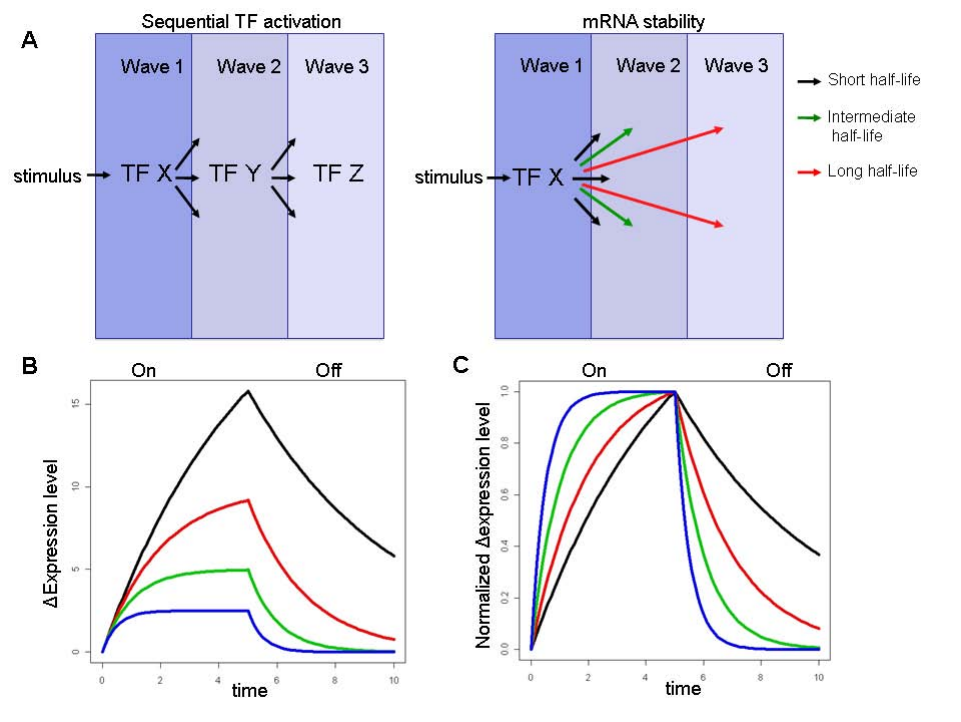
**Kinetics of gene induction**. (A) Two mechanisms which underlie the kinetics of gene induction are sequential activation of TFs and mRNA stability. While much research attention was given to the former, the latter was overlooked by many studies. Both mechanisms act in cells in parallel, and thus, the observed dynamics of transcriptional response reflects their superposition. (In the cartoon, waves 1, 2 and 3 refer to early-, intermediate- and late- kinetic responses.) (B) The standard kinetic model predicts that the rapidity of a transition between former and new transcript steady states is determined by the transcript's stability (T_1/2 _= ln2/α). The figure shows simulated kinetic response of four mRNAs with the same transcription rate (β = 5) and different degradation rates (blue: α = 2.0; green: α = 1.0; red: α = 0.5; black: α = 0.2). A pulse stimulation was exerted at t = 0 and terminated at t = 5. Note that upon induction, the most unstable mRNA (blue, highest α) reaches the lowest steady-state level, but it does so very rapidly (lowest T_1/2_). (C) Transcription rates of the four mRNAs were adjusted to bring them to the same level at t = 5.

Time-course analysis is a very common design for microarray analysis, which allows researchers to follow the dynamics of the cellular response to perturbations. Clustering analysis applied to time-course data partitions the response into distinct kinetic waves, distinguishing between early-, intermediate- and late- responding genes. Such kinetic waves can be the result of sequential activation of primary and secondary transcription factors (TFs) [8,9]. Nevertheless, mathematical modeling of changes in RNA levels predicts that mRNA degradation rate plays a pivotal role in shaping the kinetics of genes' response [10,11]. A standard measure for the speed of a transition between two steady states is T_1/2_, which is the time at which half of the change between the new and the former steady-state levels is achieved. (In the phase of mRNA decay T_1/2 _is usually referred to as 'T half-life' which measures the period of time it takes for a transcript undergoing decay to decrease its level by half). Importantly, a simple kinetic model predicts that T_1/2 _is determined by RNA degradation rate not only in decay of expression but also in the phase of induction. The model assumes that mRNA is produced at a constant rate (β) while the rate of its degradation is proportional to its concentration. Accordingly, the rate of change in mRNA concentration (X) is given by the equation: dX/dt = β-αX (α denotes the degradation rate constant). At steady state mRNA concentration reaches equilibrium (that is, there is no change: dX/dt = 0), and therefore, the steady state level (X_ss_) is determined by the ratio between the synthesis and degradation constants: X_ss _= β/α. Solving the above equation gives the change in mRNA concentration over time: ΔX(t) = [β/α-X_0_]*(1-e^-αt^) (X_0 _represents the mRNA concentration at t0 where the perturbation was applied to the system, assuming that change in transcription rate occurs instantaneously). Of note, according to this solution, the rapidity of a transition between former and new steady states (T_1/2_) is determined only by α, and is inversely proportional to it: T_1/2 _= ln2/α; Therefore, genes with unstable RNA are predicted to respond in fast kinetics, whereas genes with stable RNA should respond more slowly (Figure 1; For a thorough discussion of the kinetic model see [10]). Furthermore, this model predicts that the time it takes a transcript whose transcription rate is increased by a factor L to achieve a k-fold induction in expression is a linear function of T_1/2_: T_k _= -log_2_(1-f)*T_1/2_; where f = (k-1)/(L-1) [12]; That is, for a similar increase in transcription rate in response to a stimulus, genes encoding unstable mRNAs are predicted to achieve a certain fold of induction faster than genes which encode stable transcripts.

Recently, the critical role for mRNA stability in the induction kinetics of genes encoding inflammatory proteins was demonstrated at single-genes level [13]. Here, we set out to examine this role on a large-scale utilizing a global atlas of mRNA stability recently generated in mammalian cells and analyzing numerous gene expression time-course datasets that collectively cover many different aspects of the cellular physiology.

## Results

### Relationship between mRNA stability and induction kinetics in response to IL2

**Figure 2:**
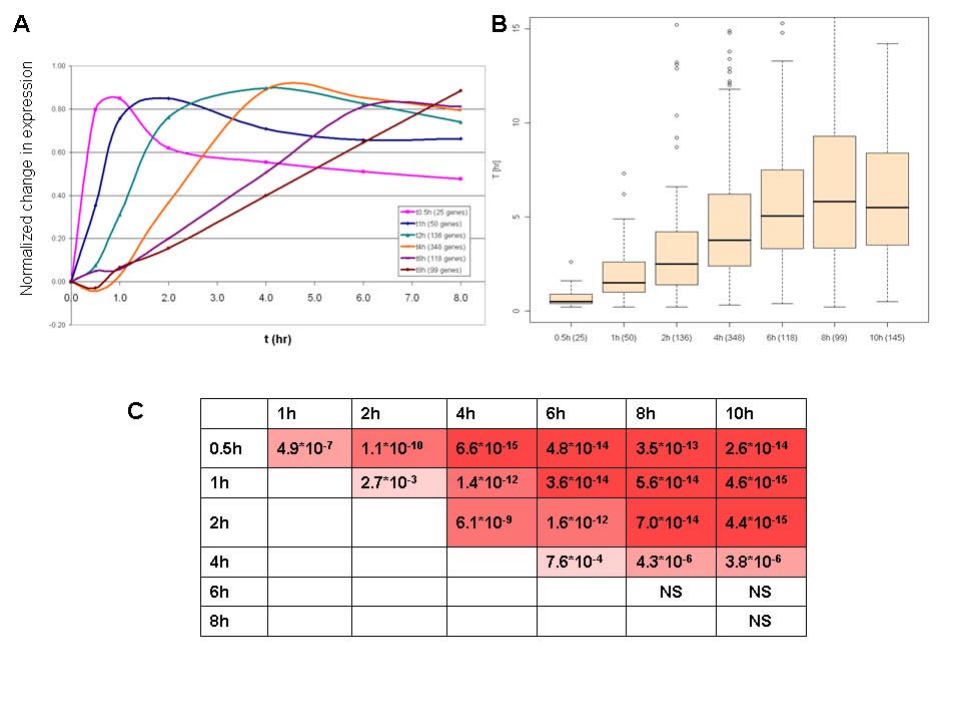
**Kinetics of gene induction in response to IL2**. (A) Kinetic clusters of genes induced by IL2 treatment. Genes were divided into clusters according to the first time in which they were induced by at least 2.0-fold (numbers of genes in each cluster are depicted in inset). Mean expression patterns of the clusters are shown. To bring genes to a similar scale, for each gene, relative expression levels to t0 (in log scale) were normalized to the gene's maximal fold of induction. After such manipulation, genes with similar kinetics but different magnitude of response show similar pattern. (B) Comparison of T half-life distribution between the different kinetic clusters. (At the X-axis, the number of genes assigned to each kinetic cluster is indicated next to the time point indicator. (C) P-values (Wilcoxon test) for the comparison between each pair of clusters. T half-life distribution of early-induced genes was significantly lower than those of genes that were induced at slower pace.

To examine the role of mRNA stability in determining the dynamics of transcriptional networks in mammalian cells, we scanned expression microarray repositories for time-course datasets that profiled cellular transcriptome in human and murine cells at dense kinetics. As a test case, we first analyzed a dataset that recorded expression profiles at multiple time points (0, 0.5, 1, 2, 4, 6, 8, and 10 hrs) after stimulation of murine T cells with IL2 [14]. We partitioned the genes that were induced by this treatment into distinct kinetic clusters according to their response time (i.e., according to the first time point in which a gene was induced above a certain fold-change threshold) (Figure 2A). A clear distinction between early-, intermediate- and late- responding genes emerged.

Next, we tested if there is a relationship between the observed kinetics of gene induction and mRNA stability. Namely, we tested whether fast responding genes were characterized by high instability (low T half-life), while late responders were more stable (high T half-life). In these tests we utilized a global atlas of mRNA stability in mammalian cells that was recently generated in murine fibroblast and human B cells [2,12]. Importantly, these reports noted that mRNA half-life times were generally well conserved between the examined cell types and species (the median T_1/2 _in human and mouse cells was 315 min and 274 min, respectively). Using this data source, we found a striking correlation between response time and transcript stability: T half-lives of early-induced genes were significantly shorter than those of late-induced genes (Figure 2B, C), as predicted by the kinetic model.

### Global relationship between mRNA stability and induction kinetics

**Figure 3:**
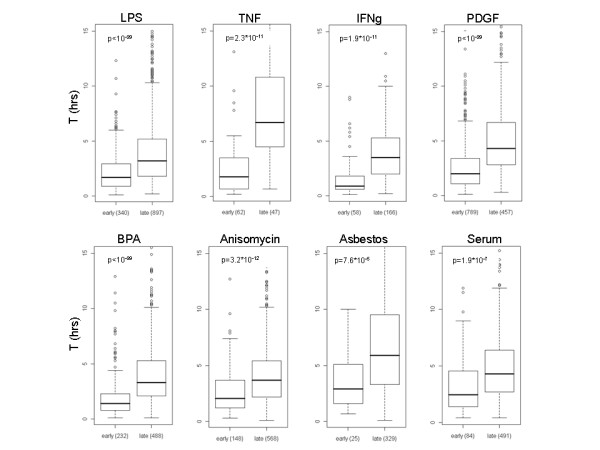
**Global relationship between mRNA stability and kinetics of gene induction**. For each dataset described in Additional file 1, we compared the T half-life distribution between early- and late- induced genes (that is, between genes that responded above the fold-change threshold specified in Additional file 1 before or at the 2 h time point and those that were induced later than 2 h). Numbers of early- and late-induced genes in each dataset are specified below the respective box-plots. (p-values were calculated using Wilcoxon test).

Next, we examined the generality of the association between kinetics of induction and RNA stability. We analyzed a variety of time-course expression datasets recorded in human and murine cells, collectively covering many different aspects of cellular physiology. In the vast majority of the datasets analyzed, we found a highly significant anti-correlation between rapidity of induction and mRNA stability (Figure 3, Additional file 1, Additional file 2). This widespread relationship points to the critical role played by mRNA stability in shaping the dynamics of gene induction in complex transcriptional networks. It also indicates a broad conservation of RNA stability under different conditions, which therefore reflects, to a large extent, an intrinsic property of the mRNA molecules. Inspection of the early-induced genes revealed a core set of genes whose induction-response was very rapid in many different datasets and which encode highly unstable transcripts (e.g., Fos, Jun, Ier3, Dusp1, Atf3, Btg2 and Zfp36; all have T half-life lower than 1 hr). (Additional file 3 lists the core set genes, defined as the set of genes that were induced before or at 2 hrs after stimulation in at least three of the six datasets recorded in murine cells.) However, the broad relationship between mRNA stability and induction kinetics is not merely explained by this common core set of rapidly induced genes, as the relationship remained highly significant also after the removal of this core set from the analysis (Additional file 4, data not shown). This indicates that many other genes with an unstable mRNA were rapidly induced in a stimulus-specific manner.

mRNA stability is regulated mainly by cis-regulatory elements embedded in the transcript 3'-UTR [5,15]. Seeking for major mechanisms that control stability in the datasets that we analyzed, we searched for enriched sequence patterns in the 3'-UTRs of the core set of early-induced genes. In agreement with previous reports [15,16], we found that these highly unstable mRNAs are significantly enriched for the AU-rich element (ARE); the most highly enriched 7-mer in the 3'-UTRs of these genes was UAUUUAU, which appeared in 52% of the 3'-UTRs in this set compared to background frequency of 18% in all 3'-UTRs of mouse genes (p-value = 7.6*10^-9^, hypergeometric tail).

### Relationship between genomic transcribed length and induction kinetics

**Figure 4:**
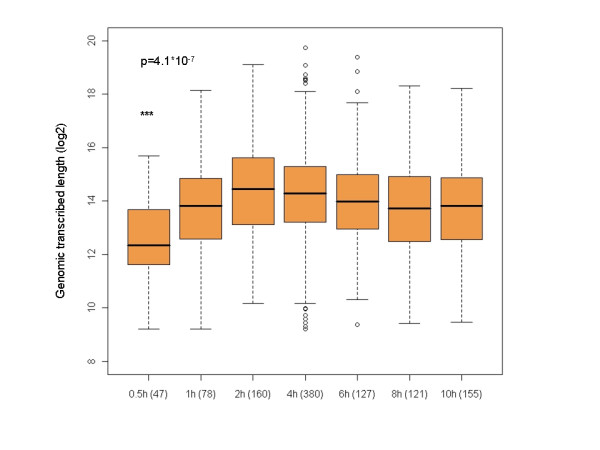
**Correlation between response time and genomic transcribed length**. Genes were divided into kinetic clusters according to the first time in which they were induced in response to IL2 (as shown in Figure 2), and distribution of genomic transcribed length was calculated for each cluster. The genes induced at 0.5 h were significantly shorter than all other induced genes (p = 4.1*10^-7^, Wilcoxon test).

Another physical factor which limits the rapidity at which genes are induced is the genomic transcribed length. The earliest time in which a transcript can be induced is bound by the length of its encoding gene and the velocity at which the RNA polymerase elongates along it. Therefore, we expected that genes which are induced at a very rapid kinetics would be characterized by short genomic transcribed length. Indeed, in the vast majority of the datasets that we analyzed we detected a significant correlation between rapidity of induction and genomic transcribed length (Figure 4, Additional file 1 and Additional file 5). However, in contrast to mRNA stability that affected the induction kinetics over a wide range of time points, the effect of genomic transcribed length was noticeable only in the very early time points after stimulation (compare Figure 2B and Figure 4). Furthermore, no correlation was observed between response time and length of mature transcripts (that is, the length of mRNA transcripts after introns are spliced out; data not shown), and there was no overall correlation between mRNA stability and genomic transcribed length (data not shown). Yet, the core set of early induced genes was characterized by both very short T half-lives and very short genomic transcribed length (Additional file 6). Therefore, to achieve very rapid gene induction, a combined strategy that couples substantial increase in transcriptional rate with rapid degradation rate and short genomic transcribed length is undertaken. Of note, this core set is enriched for TFs (16 out of 52 genes, p = 4.7*10^-5 ^after FDR correction), as agile regulation of the regulators is a critical property of networks.

### Relationship between mRNA stability and kinetics of gene suppression

The mathematical kinetic model also predicts a similar relationship between RNA stability and kinetics of gene suppression. Genes whose expression is down-regulated rapidly are expected to encode RNAs with lower stability than genes whose expression is down-regulated at slower rate (Figure 1). Interestingly, while in several datasets we observed a very good agreement with this expectation, deviations from it were frequent. In five out of the ten datasets that we analyzed, we observed no relationship between mRNA stability and kinetics of gene suppression, while this relationship was highly significant for the induced genes (Additional file 1, Additional file 7). In those cases, the expression of many mRNAs which have high T half-life under standard conditions (above 5 hrs) was already down-regulated by at least a factor of 2.0 after only 1-2 hrs post treatment. Such a rapid down-regulation of stable RNAs cannot be explained even by a complete shut-off of transcription, and therefore suggests intense modulation of mRNA stability of the rapidly suppressed genes. We could not detect statistically enriched sequence motifs in the 3'-UTR of these genes that might point to the mechanism (e.g., microRNA, RNA-binding protein) which controls this stability modulation.

## Discussion

Two mechanisms which underlie the temporal order of gene induction are sequential activation of primary and secondary TFs and mRNA stability. These mechanisms operate in parallel, and therefore the observed induction kinetics is the result of their superposition. The kinetic model we used in our study is oversimplified as it assumes constant rates of transcription and degradation over time. In practice, however, most genes are regulated by modules of transcriptional activators and repressors rather than by single TF, the activity of TFs themselves is modulated over time and they often form interlocked feedback loops. Therefore, the kinetic patterns exhibited by responding genes are much more complicated than the simple exponential pattern predicted by the model. Yet, much insight can be gained on the dynamics of transcriptional networks using the simplified description. While many studies delineated the way various transcriptional networks are propagated by sequential cascades of TFs ([9,17,18], much less attention was given to the role of mRNA stability in shaping the induction dynamics. Recently, Hao and Baltimore [13] showed that mRNA stability significantly influences the induction kinetics of genes encoding inflammatory proteins. Here, by analyzing numerous gene expression datasets that collectively cover many different aspects of cellular physiology, we demonstrated on a global scale, the critical role that mRNA stability plays in shaping the dynamics of transcriptional networks. This global relationship agrees with the simple kinetic model for mRNA concentration which predicts that unstable transcripts respond faster than stable ones.

Of note, the striking anti-correlation that we detected in a diverse panel of cell types between mRNA stability and induction kinetics was derived using an atlas of mRNA half-lives measured in murine fibroblasts and human B-cells. This result indicates a broad conservation of RNA stability under different cell types and conditions, which therefore reflects, to a large extent, an intrinsic property of the mRNA molecules. This observation supports and strengthens Friedel et al. conclusion that mRNA stability is largely conserved between cell types and species [12].

Another factor which limits gene induction time is the genomic transcribed length. In accord, we observed that genes that were induced very rapidly were significantly short. Therefore, to achieve very rapid gene induction, substantial increase in transcriptional rate is coupled with rapid degradation rate and short genomic transcribed length, as exhibited by the core set of early-induced genes.

Interestingly, while we observed very good agreement with the kinetic model when analyzed gene induction, we found major deviations from the model predictions when analyzed gene suppression. In response to many stimuli, we observed very fast down-regulation of mRNAs whose half-life time is very high under normal conditions (as measured in fibroblasts (mouse) and B-cells (human)). Even a complete turn-off of transcription is not enough to achieve such a rapid reduction in the concentration of stable mRNAs. Therefore, those mRNAs that need to be down-regulated at a very high speed require regulatory mechanisms that decrease their stability. Key regulators of mRNA stability are RNA binding proteins (RBPs) and microRNAs (miRs), and ample information on the activation of these regulators in response to various stresses has been already accumulated. We speculate that activation of stimulus-specific RBPs and miRs is a major factor that underlies the deviation from model's prediction in the case of gene suppression.

## Conclusion

Comprehensive understanding of gene expression networks can be gained only once we obtain a global delineation of the orchestrated modulation of transcription and degradation rates carried out by cells in normal development and in response to perturbations. In this study, we elucidated the key role of mRNA stability in shaping the kinetics of gene induction in intricate gene networks in mammalian cells.

## Methods

### Gene expression data analysis

Expression data were downloaded from public repositories (GEO and ArrayExpress). All datasets used Affymetrix arrays. Expression levels were calculated using the rma method [19] (implemented in Affymetirx Expression Console tool). For each dataset, presence flags were calculated using MAS5, and only probe-sets that were flagged as 'Present' in at least two chips were retained for subsequent analysis. For genes represented by multiple probe-sets, we chose the one probe-set with the highest median intensity in the dataset.

Kinetic clustering and all statistical analyses of T half-life and genomic transcribed length distributions were done in R.

### Transcript sequences and genomic transcribed length

Transcript sequences and lengths for all human and mouse genes were obtained using Biomart (Ensembl v54) [20]. For genes encoding multiple transcripts, the transcript with the longest genomic transcribed length was used in length distribution tests.

### Enrichment tests

Enrichment test for k-mers in 3'-UTR of murine genes was carried out using the AMADEUS tool [21]. For genes with multiple forms of 3'-UTR, the longest one was used. Enrichment test for GO functional categories was carried out using DAVID web-service [22]. In both, the core set of early-induced genes was compared to a background set of all murine genes.

## Authors' contributions

RE and RA conceived and designed the study. RE carried out the statistical analyses. EZ and KZ performed gene expression experiments. RE and RA wrote the manuscript. All authors read and approved the final manuscript.

## Supplementary Material

Additional file 1 Relationship between mRNA stability, response kinetics and genomic transcribed length in ten expression datasets. Click here for file

Additional file 2 **Relationship between mRNA stability and kinetics of induction in various datasets (see legend of **Figure 2B, C**and **Additional file 1). Click here for file

Additional file 3 Core set of early-induced genes. Click here for file

Additional file 4 **Examination of the relationship between mRNA stability and kinetics of induction in the IL2 dataset after removing from the analysis the core set of early induced genes**. p-value was calculated for the comparison between the distribution of T half-life of early and late induced genes, as done in Additional file 1, but after the removal of the core genes. Click here for file

Additional file 5 **Relationship between kinetics of induction and genomic transcribed length**. The effect of genomic transcribed length on the response time is evident only at the very early time points (up to 1-2 hrs after stimulation; see legend of Additional file 1). Click here for file

Additional file 6 **The core set of early induced genes is characterized by both (a) very short T half-life (mean T_1/2 _of 0.90 h vs. 6.45 h, in the core and background sets, respectively) and (b) very short genomic transcribed length (mean genomic transcribedlength of 9,474 bp vs. 39,789 bp, in the core and background sets, respectively).** P-values (Wilcoxon test) were calculated for the comparison between the core set and a background set which contained all the rest of genes for which T half-life and genomic transcribed length (i.e., CDS and UTRs annotations) data are available. Click here for file

Additional file 7 **Relationship between mRNA stability and kinetics of gene repression in various datasets**. Deviations from model prediction are much more frequent here than in the analysis of gene induction (compare with Additional file 2). Click here for file

## References

[B1] Garcia-Martinez J Aranda A Perez-Ortin JE Genomic run-on evaluates transcription rates for all yeast genes and identifies gene regulatory mechanisms Molecular cell 2004 15 2 303 313 10.1016/j.molcel.2004.06.004 15260981

[B2] Dolken L Ruzsics Z Radle B Friedel CC Zimmer R Mages J Hoffmann R Dickinson P Forster T Ghazal P High-resolution gene expression profiling for simultaneous kinetic parameter analysis of RNA synthesis and decay RNA (New York, NY) 2008 14 9 1959 1972 10.1261/rna.1136108 PMC2525961 18658122

[B3] Cheadle C Fan J Cho-Chung YS Werner T Ray J Do L Gorospe M Becker KG Control of gene expression during T cell activation: alternate regulation of mRNA transcription and mRNA stability BMC genomics 2005 6 1 75 10.1186/1471-2164-6-75 15907206 PMC1156890

[B4] Keene JD RNA regulons: coordination of post-transcriptional events Nature reviews 2007 8 7 533 543 10.1038/nrg2111 17572691

[B5] Narsai R Howell KA Millar AH O'Toole N Small I Whelan J Genome-wide analysis of mRNA decay rates and their determinants in Arabidopsis thaliana The Plant cell 2007 19 11 3418 3436 10.1105/tpc.107.055046 18024567 PMC2174890

[B6] Romero-Santacreu L Moreno J Perez-Ortin JE Alepuz P Specific and global regulation of mRNA stability during osmotic stress in Saccharomyces cerevisiae RNA (New York, NY) 2009 15 6 1110 1120 10.1261/rna.1435709 PMC2685517 19369426

[B7] Shalem O Dahan O Levo M Martinez MR Furman I Segal E Pilpel Y Transient transcriptional responses to stress are generated by opposing effects of mRNA production and degradation Molecular systems biology 2008 4 223 10.1038/msb.2008.59 18854817 PMC2583085

[B8] Bolouri H Davidson EH Transcriptional regulatory cascades in development: initial rates, not steady state, determine network kinetics Proceedings of the National Academy of Sciences of the United States of America 2003 100 16 9371 9376 10.1073/pnas.1533293100 12883007 PMC170925

[B9] Smith J Theodoris C Davidson EH A gene regulatory network subcircuit drives a dynamic pattern of gene expression Science (New York, NY) 2007 318 5851 794 797 10.1126/science.1146524 17975065

[B10] Perez-Ortin JE Alepuz PM Moreno J Genomics and gene transcription kinetics in yeast Trends Genet 2007 23 5 250 257 10.1016/j.tig.2007.03.006 17379352

[B11] Barenco M Tomescu D Brewer D Callard R Stark J Hubank M Ranked prediction of p53 targets using hidden variable dynamic modeling Genome biology 2006 7 3 R25 10.1186/gb-2006-7-3-r25 16584535 PMC1557743

[B12] Friedel CC Dolken L Ruzsics Z Koszinowski UH Zimmer R Conserved principles of mammalian transcriptional regulation revealed by RNA half-life Nucleic acids research 2009 37 17 e115 10.1093/nar/gkp542 19561200 PMC2761256

[B13] Hao S Baltimore D The stability of mRNA influences the temporal order of the induction of genes encoding inflammatory molecules Nature immunology 2009 10 3 281 288 10.1038/ni.1699 19198593 PMC2775040

[B14] Zhang Z Martino A Faulon JL Identification of expression patterns of IL-2-responsive genes in the murine T cell line CTLL-2 J Interferon Cytokine Res 2007 27 12 991 995 10.1089/jir.2006.0169 18184039

[B15] Yang E van Nimwegen E Zavolan M Rajewsky N Schroeder M Magnasco M Darnell JE Jr Decay rates of human mRNAs: correlation with functional characteristics and sequence attributes Genome research 2003 13 8 1863 1872 12902380 10.1101/gr.1272403 PMC403777

[B16] Khabar KS The AU-rich transcriptome: more than interferons and cytokines, and its role in disease J Interferon Cytokine Res 2005 25 1 1 10 10.1089/jir.2005.25.1 15684617

[B17] Litvak V Ramsey SA Rust AG Zak DE Kennedy KA Lampano AE Nykter M Shmulevich I Aderem A Function of C/EBPdelta in a regulatory circuit that discriminates between transient and persistent TLR4-induced signals Nature immunology 2009 10 4 437 443 10.1038/ni.1721 19270711 PMC2780024

[B18] Elkon R Linhart C Halperin Y Shiloh Y Shamir R Functional genomic delineation of TLR-induced transcriptional networks BMC genomics 2007 8 394 10.1186/1471-2164-8-394 17967192 PMC2175519

[B19] Irizarry RA Bolstad BM Collin F Cope LM Hobbs B Speed TP Summaries of Affymetrix GeneChip probe level data Nucleic acids research 2003 31 4 e15 10.1093/nar/gng015 12582260 PMC150247

[B20] Haider S Ballester B Smedley D Zhang J Rice P Kasprzyk A BioMart Central Portal--unified access to biological data Nucleic acids research 2009 37 Web Server W23 27 10.1093/nar/gkp265 19420058 PMC2703988

[B21] Linhart C Halperin Y Shamir R Transcription factor and microRNA motif discovery: the Amadeus platform and a compendium of metazoan target sets Genome research 2008 18 7 1180 1189 10.1101/gr.076117.108 18411406 PMC2493407

[B22] Huang da W Sherman BT Lempicki RA Systematic and integrative analysis of large gene lists using DAVID bioinformatics resources Nature protocols 2009 4 1 44 57 10.1038/nprot.2008.211 19131956

