# Supplementary material for: Major role for mRNA stability in shaping the kinetics of gene induction
Source: BMC Genomics. 2010 Apr 21;11:259. doi: 10.1186/1471-2164-11-259 (PMC2864252; doi:10.1186/1471-2164-11-259)
Supplement: Additional file 1 — Relationship between mRNA stability, response kinetics and genomic transcribed length in ten expression datasets. [file 1471-2164-11-259-S1.DOC]

**Additional File1. Relationship between mRNA stability, response kinetics and genomic transcribed length**

| **Stimulation/**  **Stress** | **Dataset ID** | **Species** | **Cell type** | **Time points** | **FC thresh^§^** | **P value - Induction^#^** | **P value – Down regulation^##^** | **P value – genomic length^###^** |
| --- | --- | --- | --- | --- | --- | --- | --- | --- |
| IL2 [1] | GSE6085 | Mm | T cells | 0.5, 1, 2, 4, 6, 8, 10 hrs | 2.0 | <10^-99^ | 6.13*10^-9^ | 4.1*10^-7^ |
| LPS [2] | GSE14769 | Mm | Macrophage | 20, 40, 60, 80 min, 2, 4, 6 hrs | 1.7 | <10^-99^ | <10^-99^ | <10^-99^ |
| TNF [3] | GSE14071 | Mm | Embryonic fibroblasts | 0.5, 2, 12 hrs | 1.7 | 2.3*10^-11^ | 4.8*10^-5^ | 2.0*10^-7^ |
| IFN gamma [4] | E-MEXP-1490 | Mm | Bone marrow macrophage | 1, 2, 4, 8 hrs | 2.0 | 1.9*10^-11^ | 0.03 | 3.3*10^-12^ |
| PDGF [5] | GSE6526 | Mm | Smooth muscle | 15, 30, 60, 120 min, 3, 4, 8 hrs | 1.7 | <10^-99^ | <10^-99^ | 3.8*10^-10^ |
| Bisphenol A (BPA) [6] | GSE4650 | Mm | Testicular Sertoli cells | 3, 6, 12 hrs | 1.7 | <10^-99 *^ | NS | 4.8*10^-7^ |
| Anisomycin [7] | GSE8229 | Hs | Myeloid leukemia cells | 1, 2, 3, 6 hrs | 1.5 | 3.2*10^-12^ | NS | <10^-99^ |
| Asbestos [8] | GSE6013 | Hs | Bronchial epithelial | 1, 6 hrs | 1.7 | 7.6*10^-5^ | NS | NS |
| IR | Unpublished^***^ | Hs | Breast epithelial | 2, 3, 4, 6, 8 hrs | 1.5 | 3.7*10^-11^ | NS | 4.7*10^-4 **^ |
| Serum | GSE20641 | Hs | Fibroblasts | 2, 4, 6, 8 hrs | 1.7 | 1.9*10^-7^ | 2.9*10^-8^ | 3.2*10^-6 **^ |

NS = not significant (p-value>0.05)

^§^ Each dataset was analyzed with three FC thresholds: 1.5, 1.7 and 2.0. Reported here is the threshold which yielded the most statistically significant relationship between induction kinetics and mRNA stability.

^#^ p-value (Wilcoxon test) for the comparison of T half-life distribution between genes induced up to 2 hrs after stimulation (“early induction”) and those induced later than 2 hrs (“late induction”).

^##^ Same as previous column, but for the comparison between early and late down-regulated genes.

^###^ p-value (Wilcoxon test) for the comparison of genomic transcribed length between genes induced up to 1 hr after stimulation and those induced later than 1 hr.

^*^ In this dataset comparisons were done between genes induced at 3 hrs and later than 3 hrs.

^**^ In these datasets genomic transcribed length comparisons were done between genes induced at 2 hrs and later than 2 hrs.

^***^ Dataset was recorded in the lab of Yosef Shiloh, Tel Aviv University.

1. Zhang Z, Martino A, Faulon JL: **Identification of expression patterns of IL-2-responsive genes in the murine T cell line CTLL-2**. *J Interferon Cytokine Res* 2007, **27**(12):991-995.

2. Litvak V, Ramsey SA, Rust AG, Zak DE, Kennedy KA, Lampano AE, Nykter M, Shmulevich I, Aderem A: **Function of C/EBPdelta in a regulatory circuit that discriminates between transient and persistent TLR4-induced signals**. *Nature immunology* 2009, **10**(4):437-443.

3. Hao S, Baltimore D: **The stability of mRNA influences the temporal order of the induction of genes encoding inflammatory molecules**. *Nature immunology* 2009, **10**(3):281-288.

4. Raza S, Robertson KA, Lacaze PA, Page D, Enright AJ, Ghazal P, Freeman TC: **A logic-based diagram of signalling pathways central to macrophage activation**. *BMC systems biology* 2008, **2**:36.

5. Shirvani SM, Mookanamparambil L, Ramoni MF, Chin MT: **Transcription factor CHF1/Hey2 regulates the global transcriptional response to platelet-derived growth factor in vascular smooth muscle cells**. *Physiological genomics* 2007, **30**(1):61-68.

6. Tabuchi Y, Takasaki I, Kondo T: **Identification of genetic networks involved in the cell injury accompanying endoplasmic reticulum stress induced by bisphenol A in testicular Sertoli cells**. *Biochemical and biophysical research communications* 2006, **345**(3):1044-1050.

7. Hori T, Kondo T, Tabuchi Y, Takasaki I, Zhao QL, Kanamori M, Yasuda T, Kimura T: **Molecular mechanism of apoptosis and gene expressions in human lymphoma U937 cells treated with anisomycin**. *Chemico-biological interactions* 2008, **172**(2):125-140.

8. Nymark P, Lindholm PM, Korpela MV, Lahti L, Ruosaari S, Kaski S, Hollmen J, Anttila S, Kinnula VL, Knuutila S: **Gene expression profiles in asbestos-exposed epithelial and mesothelial lung cell lines**. *BMC genomics* 2007, **8**:62.
