# Supplementary material for: Major role for mRNA stability in shaping the kinetics of gene induction
Source: BMC Genomics. 2010 Apr 21;11:259. doi: 10.1186/1471-2164-11-259 (PMC2864252; doi:10.1186/1471-2164-11-259)
Supplement: Additional file 2 — Relationship between mRNA stability and kinetics of induction in various datasets (see legend of Figure 2B, Cand Additional file 1). [file 1471-2164-11-259-S2.PPT]

## Slide 1
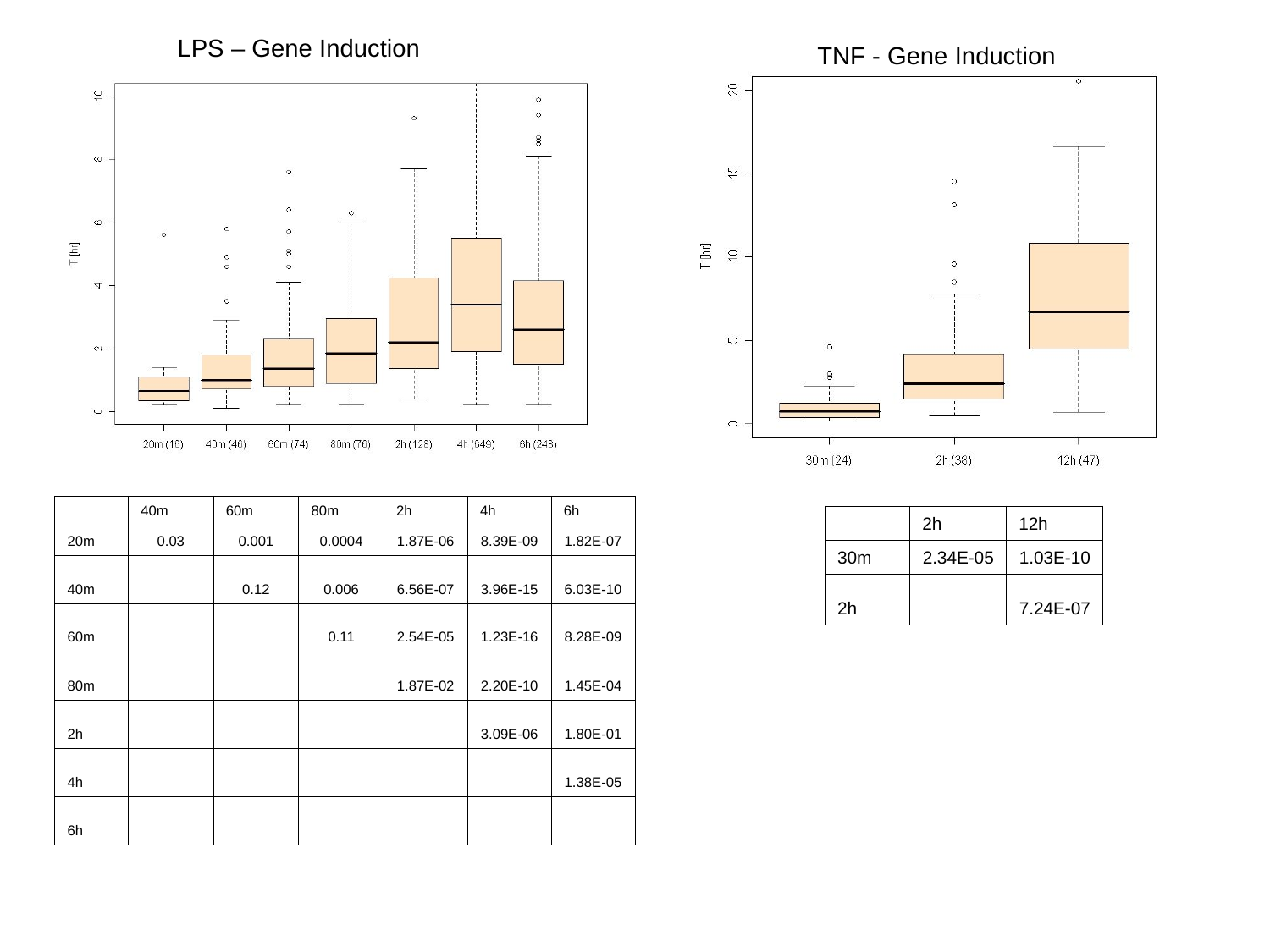

LPS – Gene Induction
TNF - Gene Induction
| | 40m | 60m | 80m | 2h | 4h | 6h |
| --- | --- | --- | --- | --- | --- | --- |
| 20m | 0.03 | 0.001 | 0.0004 | 1.87E-06 | 8.39E-09 | 1.82E-07 |
| 40m | | 0.12 | 0.006 | 6.56E-07 | 3.96E-15 | 6.03E-10 |
| 60m | | | 0.11 | 2.54E-05 | 1.23E-16 | 8.28E-09 |
| 80m | | | | 1.87E-02 | 2.20E-10 | 1.45E-04 |
| 2h | | | | | 3.09E-06 | 1.80E-01 |
| 4h | | | | | | 1.38E-05 |
| 6h | | | | | | |
| | 2h | 12h |
| --- | --- | --- |
| 30m | 2.34E-05 | 1.03E-10 |
| 2h | | 7.24E-07 |

## Slide 2
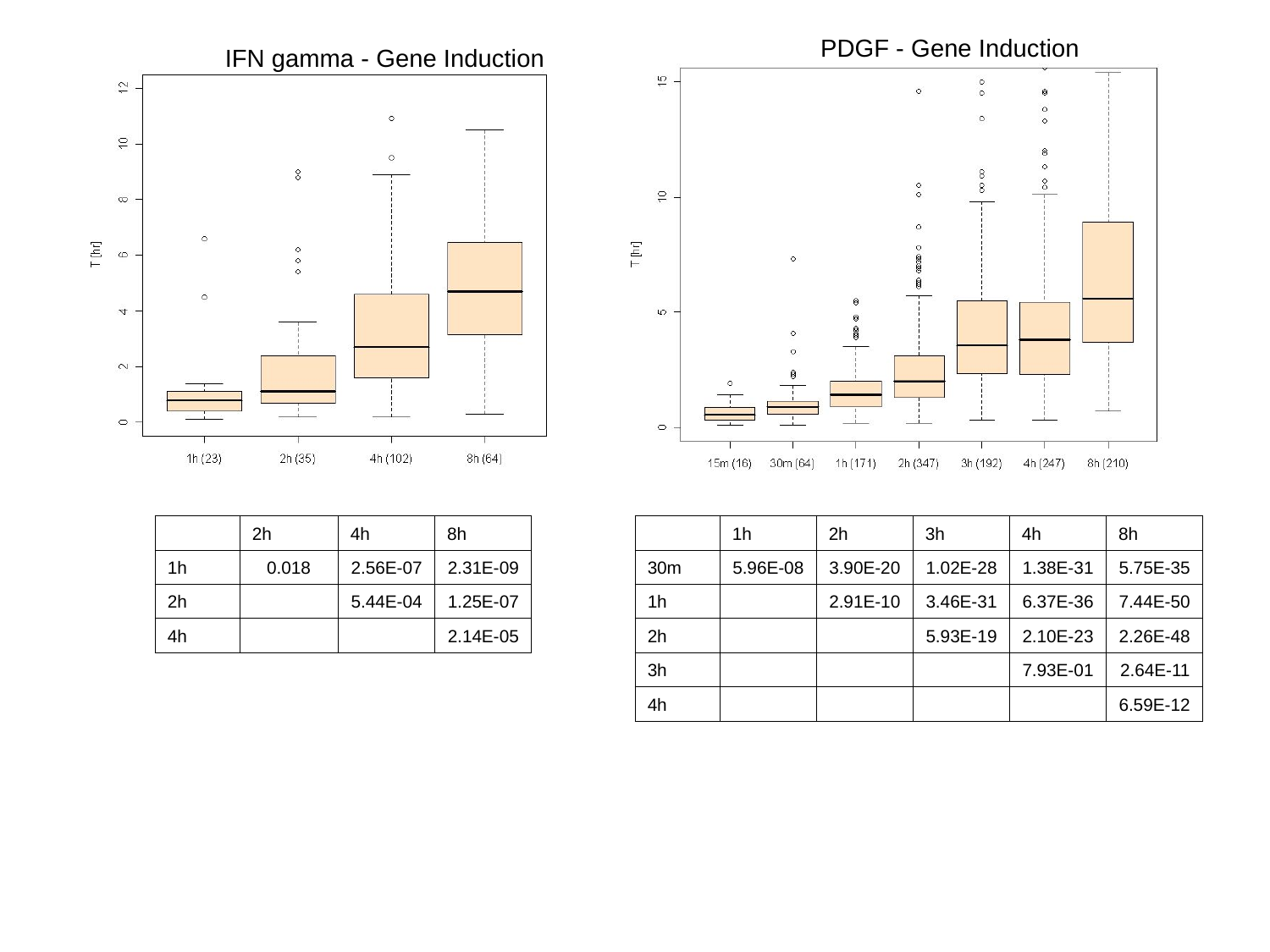

PDGF - Gene Induction
IFN gamma - Gene Induction
| | 2h | 4h | 8h |
| --- | --- | --- | --- |
| 1h | 0.018 | 2.56E-07 | 2.31E-09 |
| 2h | | 5.44E-04 | 1.25E-07 |
| 4h | | | 2.14E-05 |
| | 1h | 2h | 3h | 4h | 8h |
| --- | --- | --- | --- | --- | --- |
| 30m | 5.96E-08 | 3.90E-20 | 1.02E-28 | 1.38E-31 | 5.75E-35 |
| 1h | | 2.91E-10 | 3.46E-31 | 6.37E-36 | 7.44E-50 |
| 2h | | | 5.93E-19 | 2.10E-23 | 2.26E-48 |
| 3h | | | | 7.93E-01 | 2.64E-11 |
| 4h | | | | | 6.59E-12 |

## Slide 3
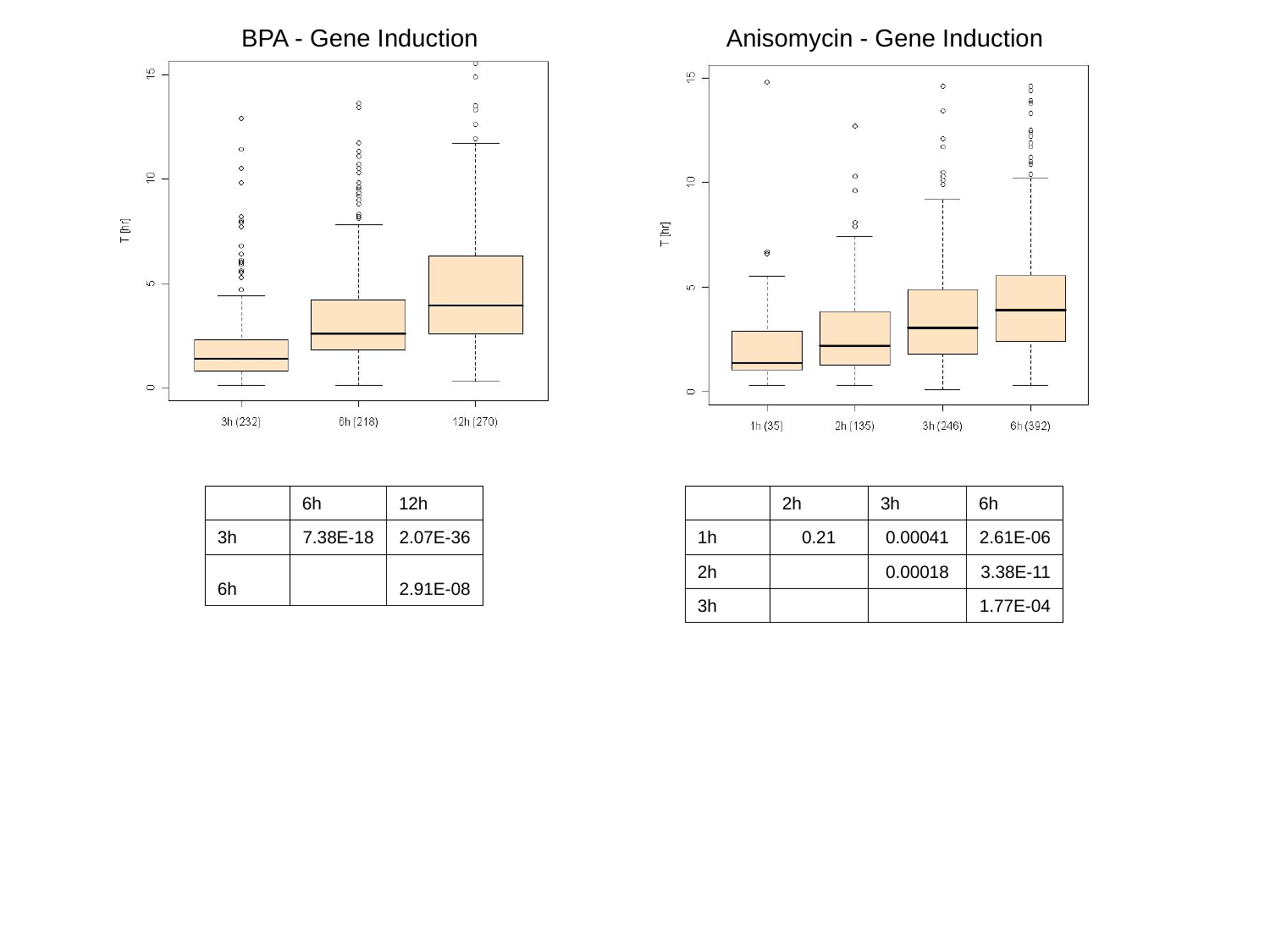

BPA - Gene Induction
Anisomycin - Gene Induction
| | 6h | 12h |
| --- | --- | --- |
| 3h | 7.38E-18 | 2.07E-36 |
| 6h | | 2.91E-08 |
| | 2h | 3h | 6h |
| --- | --- | --- | --- |
| 1h | 0.21 | 0.00041 | 2.61E-06 |
| 2h | | 0.00018 | 3.38E-11 |
| 3h | | | 1.77E-04 |

## Slide 4
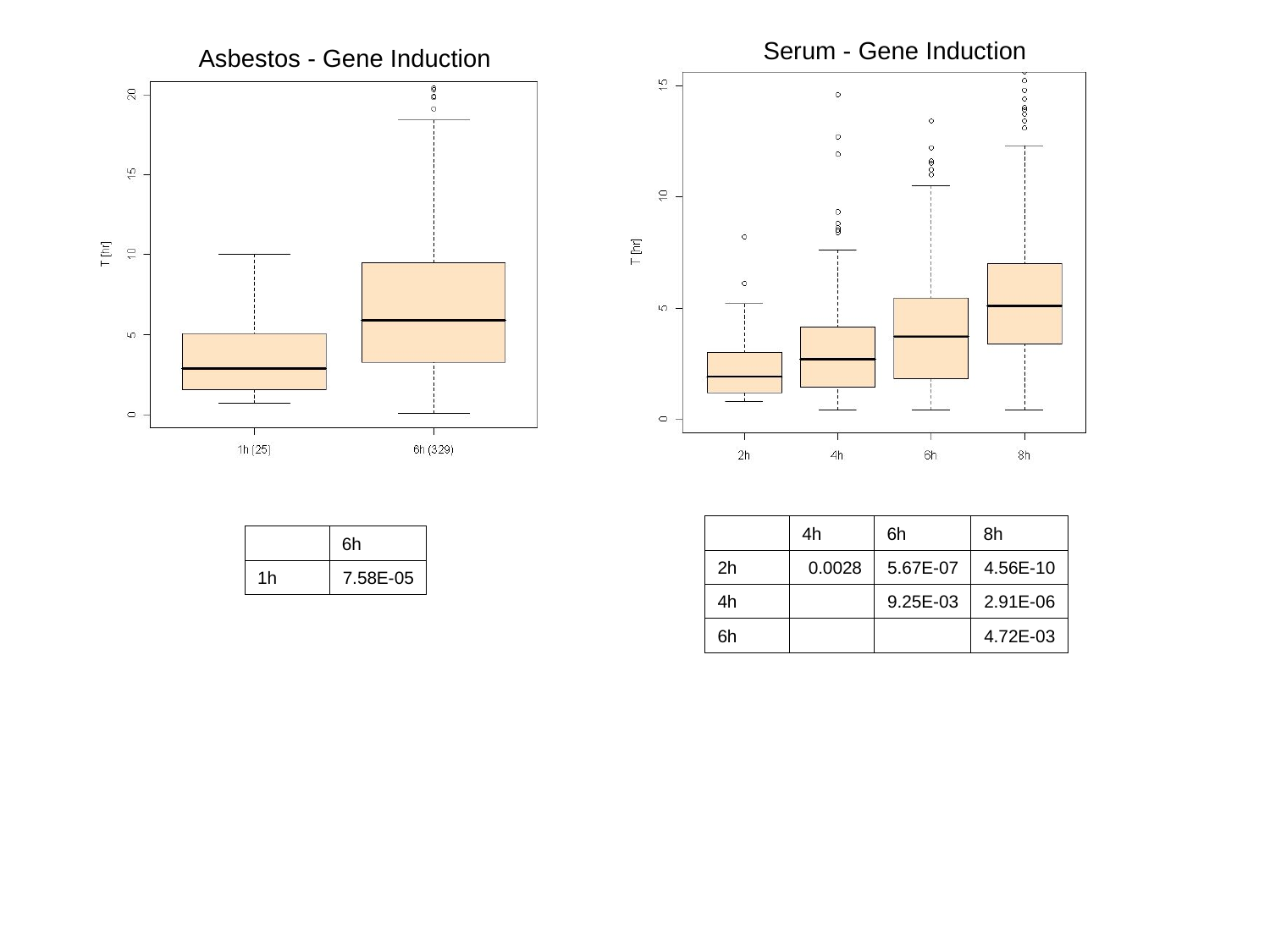

Serum - Gene Induction
Asbestos - Gene Induction
| | 4h | 6h | 8h |
| --- | --- | --- | --- |
| 2h | 0.0028 | 5.67E-07 | 4.56E-10 |
| 4h | | 9.25E-03 | 2.91E-06 |
| 6h | | | 4.72E-03 |
| | 6h |
| --- | --- |
| 1h | 7.58E-05 |
