# Supplementary figures and images for: Major role for mRNA stability in shaping the kinetics of gene induction
Source: BMC Genomics. 2010 Apr 21;11:259. doi: 10.1186/1471-2164-11-259 (PMC2864252; doi:10.1186/1471-2164-11-259)

## Slide 1
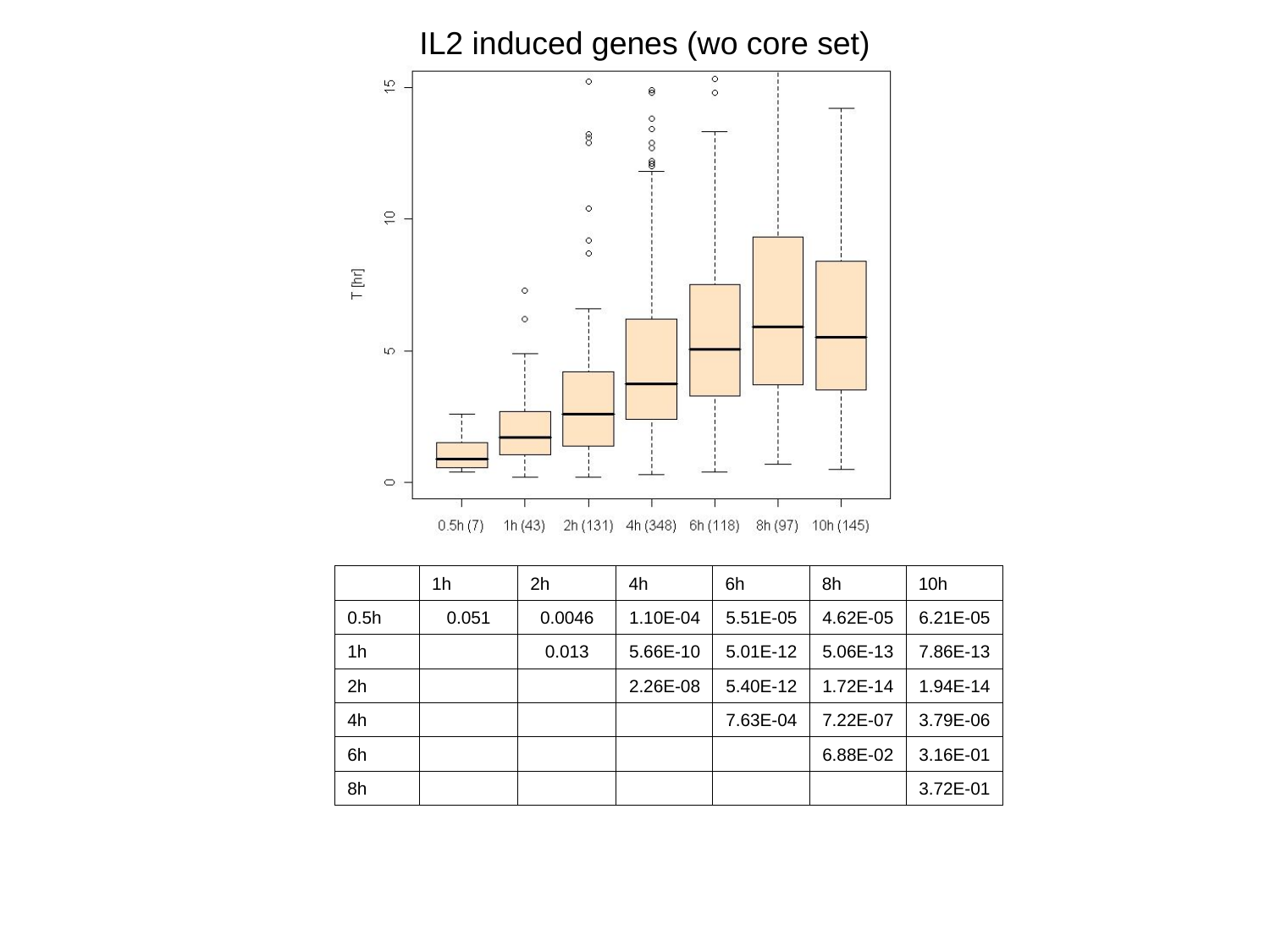

IL2 induced genes (wo core set)
| | 1h | 2h | 4h | 6h | 8h | 10h |
| --- | --- | --- | --- | --- | --- | --- |
| 0.5h | 0.051 | 0.0046 | 1.10E-04 | 5.51E-05 | 4.62E-05 | 6.21E-05 |
| 1h | | 0.013 | 5.66E-10 | 5.01E-12 | 5.06E-13 | 7.86E-13 |
| 2h | | | 2.26E-08 | 5.40E-12 | 1.72E-14 | 1.94E-14 |
| 4h | | | | 7.63E-04 | 7.22E-07 | 3.79E-06 |
| 6h | | | | | 6.88E-02 | 3.16E-01 |
| 8h | | | | | | 3.72E-01 |

Supplement: Additional file 4 — Examination of the relationship between mRNA stability and kinetics of induction in the IL2 dataset after removing from the analysis the core set of early induced genes. p-value was calculated for the comparison between the distribution of T half-life of early and late induced genes, as done in Additional file 1, but after the removal of the core genes. [file 1471-2164-11-259-S4.PPT]

## Slide 1
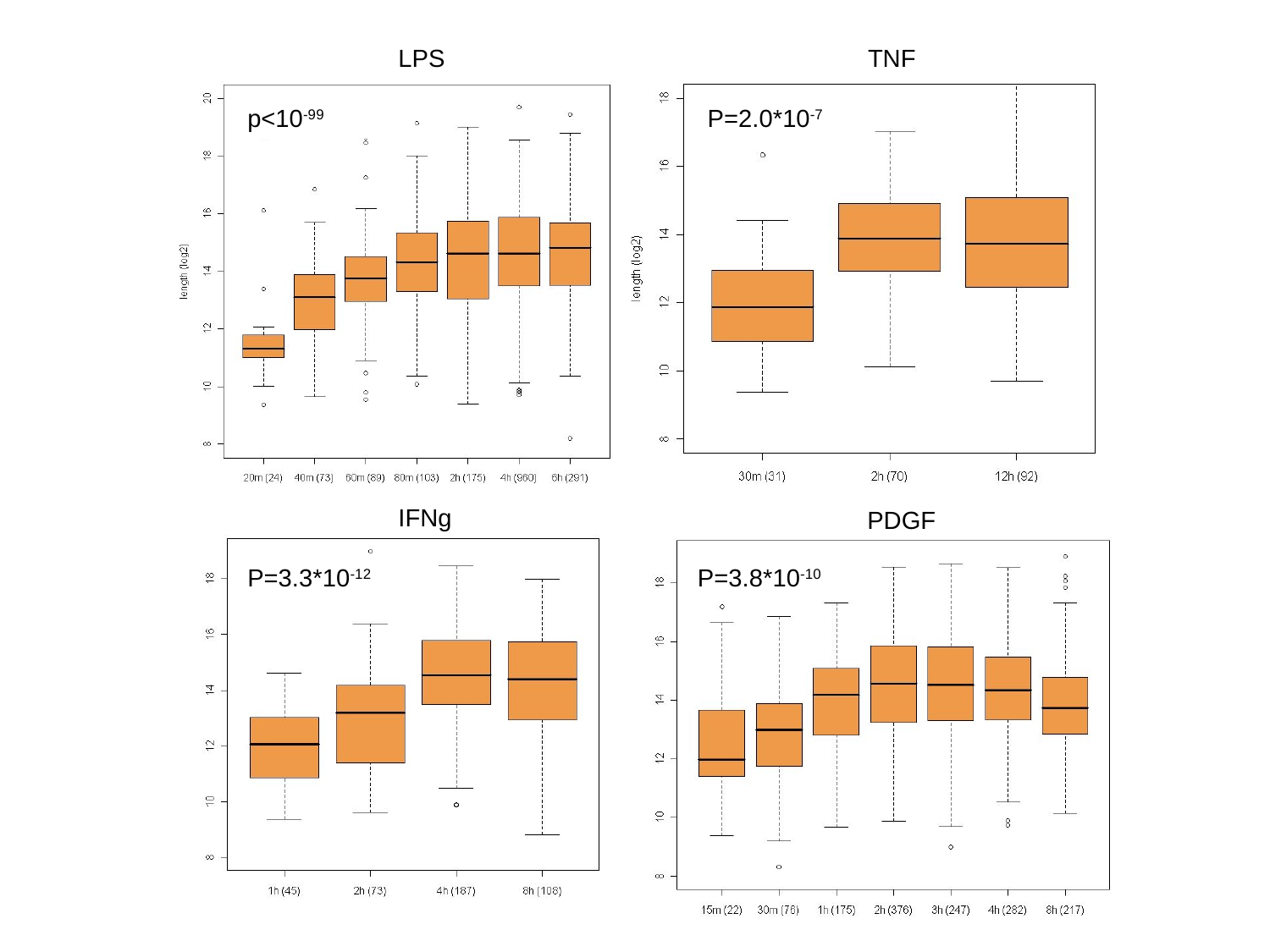

LPS
TNF
p<10-99
P=2.0*10-7
IFNg
PDGF
P=3.3*10-12
P=3.8*10-10

## Slide 2
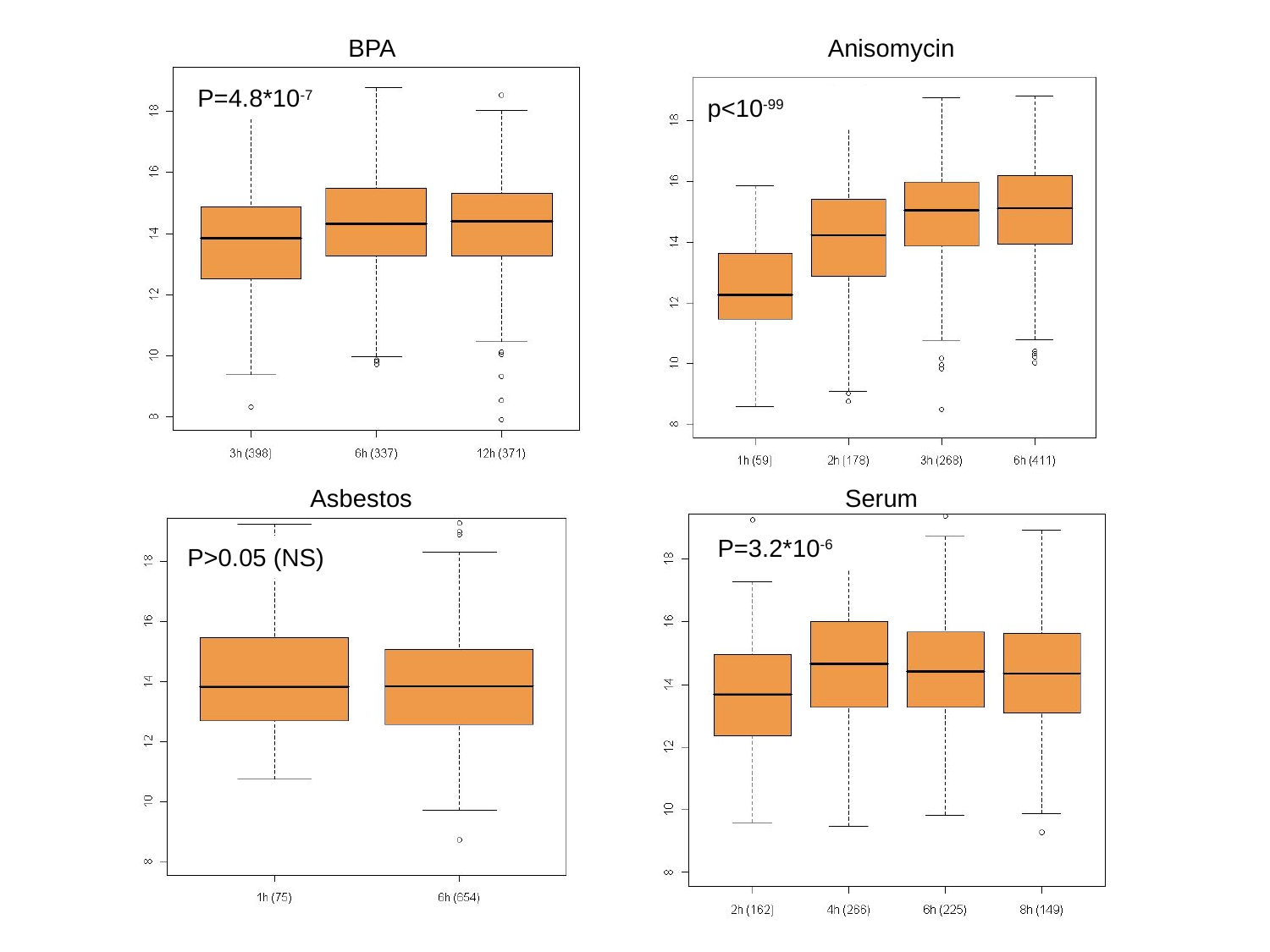

BPA
Anisomycin
P=4.8*10-7
p<10-99
Asbestos
Serum
P=3.2*10-6
P>0.05 (NS)

Supplement: Additional file 5 — Relationship between kinetics of induction and genomic transcribed length. The effect of genomic transcribed length on the response time is evident only at the very early time points (up to 1-2 hrs after stimulation; see legend of Additional file 1). [file 1471-2164-11-259-S5.PPT]

## Slide 1
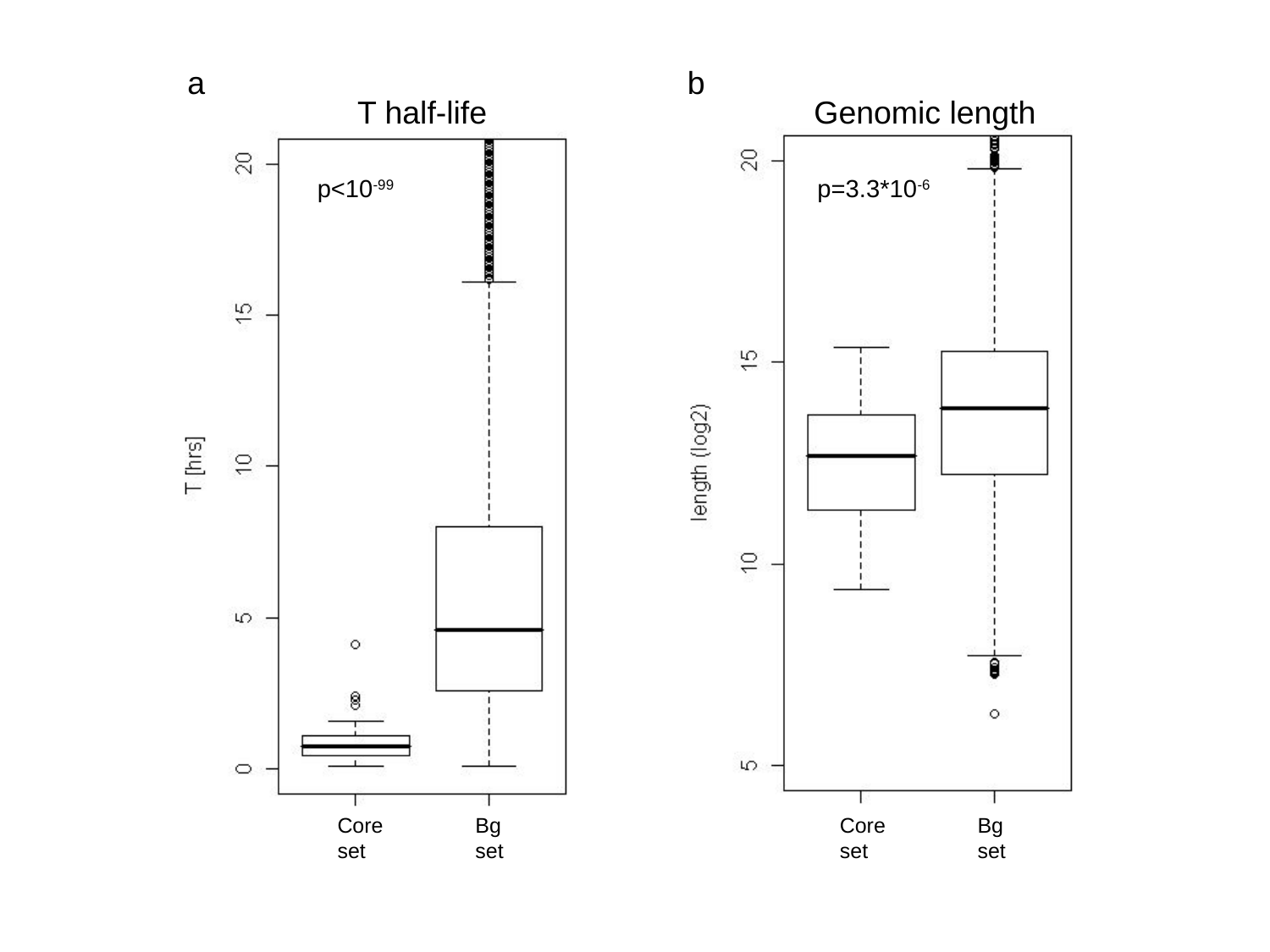

a
b
T half-life
Genomic length
p<10-99
p=3.3*10-6
Core set
Bg set
Core set
Bg set

Supplement: Additional file 6 — The core set of early induced genes is characterized by both (a) very short T half-life (mean T1/2 of 0.90 h vs. 6.45 h, in the core and background sets, respectively) and (b) very short genomic transcribed length (mean genomic transcribedlength of 9,474 bp vs. 39,789 bp, in the core and background sets, respectively). P-values (Wilcoxon test) were calculated for the comparison between the core set and a background set which contained all the rest of genes for which T half-life and genomic transcribed length (i.e., CDS and UTRs annotations) data are available. [file 1471-2164-11-259-S6.PPT]
