# Supplementary material for: Major role for mRNA stability in shaping the kinetics of gene induction
Source: BMC Genomics. 2010 Apr 21;11:259. doi: 10.1186/1471-2164-11-259 (PMC2864252; doi:10.1186/1471-2164-11-259)
Supplement: Additional file 7 — Relationship between mRNA stability and kinetics of gene repression in various datasets. Deviations from model prediction are much more frequent here than in the analysis of gene induction (compare with Additional file 2). [file 1471-2164-11-259-S7.PPT]

## Slide 1
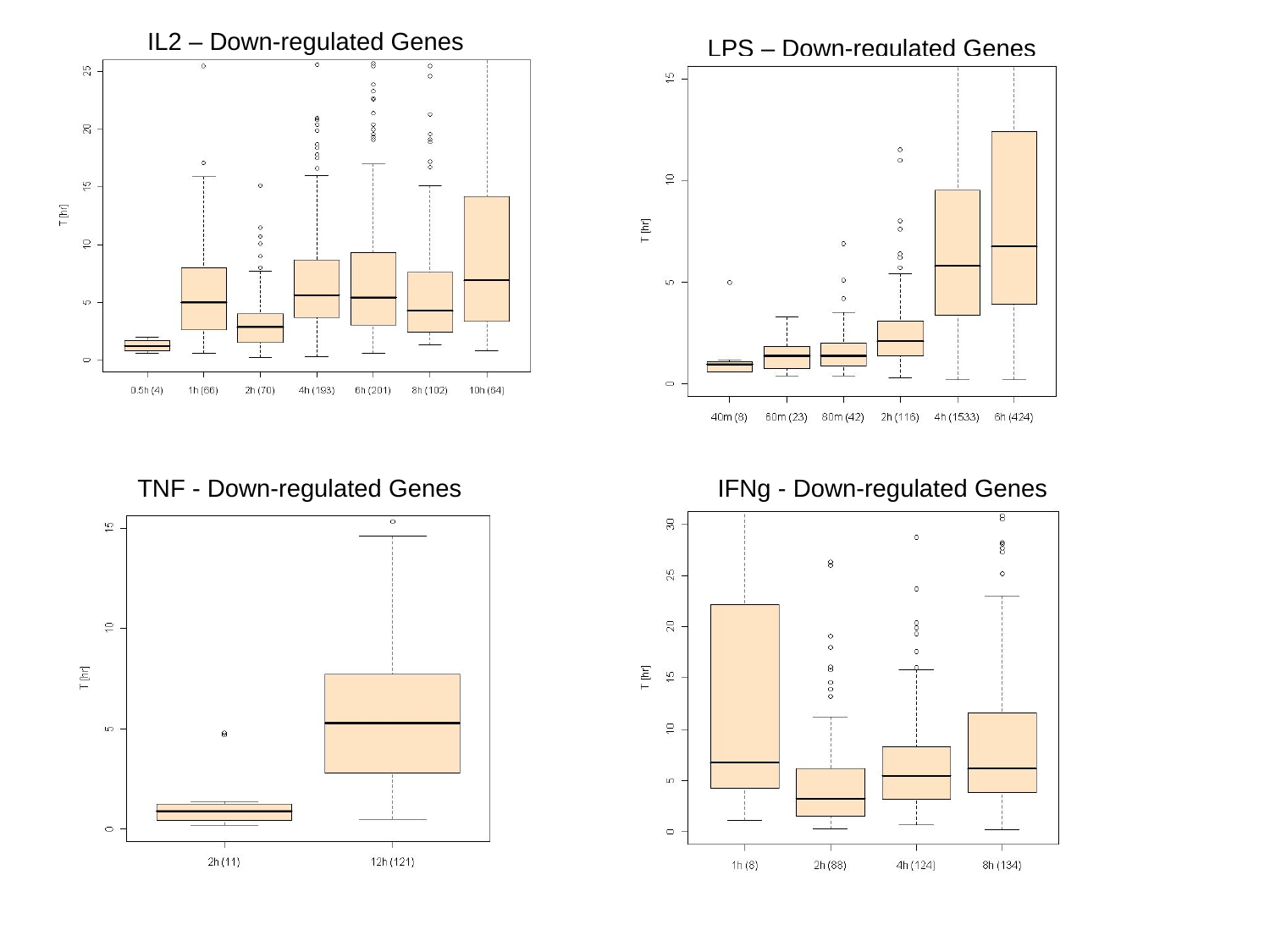

IL2 – Down-regulated Genes
LPS – Down-regulated Genes
TNF - Down-regulated Genes
IFNg - Down-regulated Genes

## Slide 2
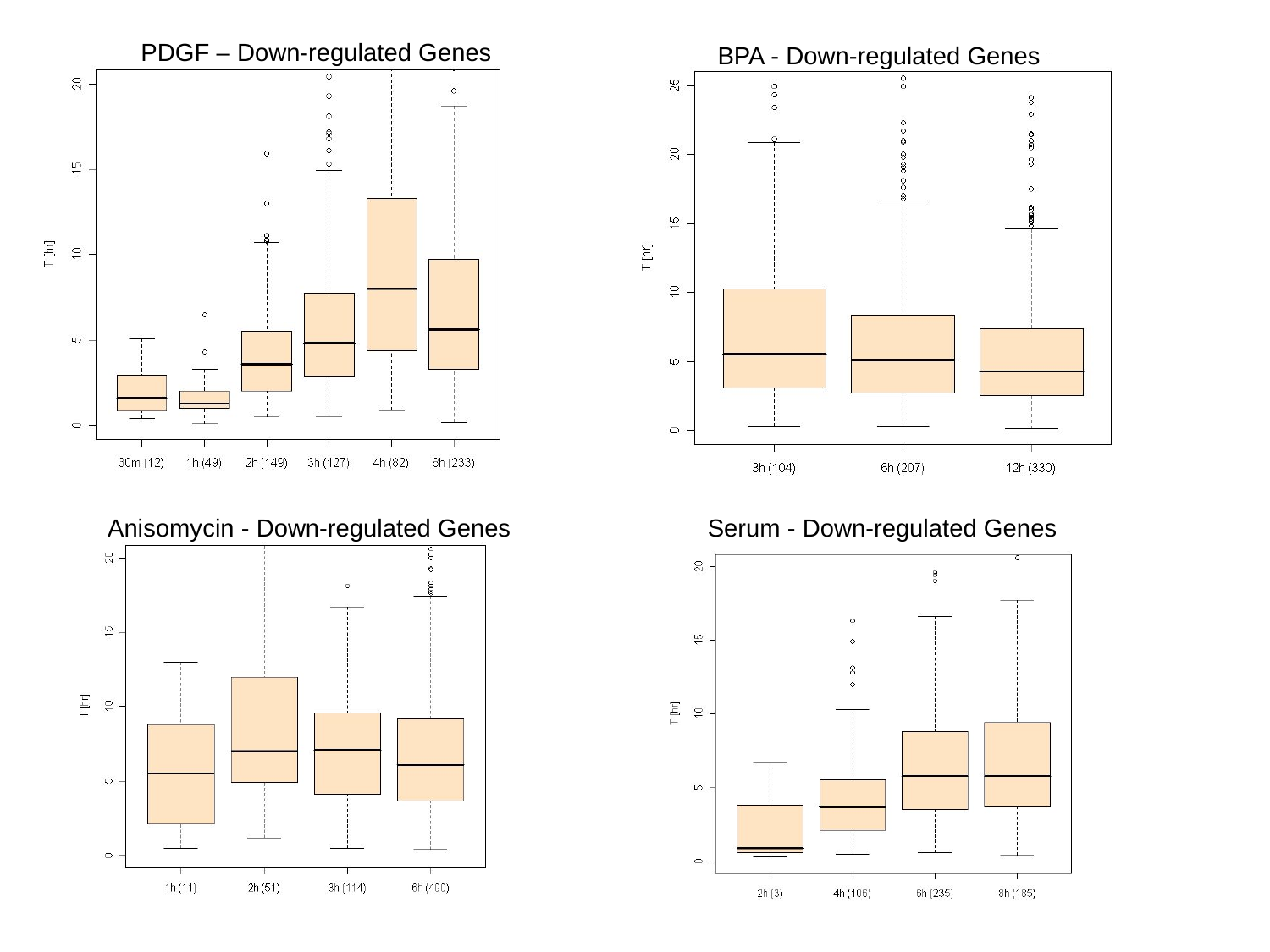

PDGF – Down-regulated Genes
BPA - Down-regulated Genes
Anisomycin - Down-regulated Genes
Serum - Down-regulated Genes
